# The relationship between a fish-rich diet and poststroke cognitive impairment: A cross-sectional study with a follow-up in China

**DOI:** 10.1097/MD.0000000000029234

**Published:** 2022-06-24

**Authors:** Jia-Rui Li, Yang Yu, Fan-Xia Meng, Jie Yu, Ben-Yan Luo, Jian Gao

**Affiliations:** a Department of Neurology and Brain Medical Center, First Affiliated Hospital, School of Medicine, Zhejiang University, Hangzhou, China; b Zhejiang University School of Medicine, Hangzhou, China; c Department of Rehabilitation, Hangzhou Mingzhou Brain Rehabilitation Hospital, Hangzhou, China.

**Keywords:** cognitive function, fish-rich diet, ischemic stroke, poststroke cognitive impairment, vascular cognitive impairment

## Abstract

Whether a fish-rich diet is positively associated with cognitive function after stroke remains unclear; thus, the present study investigated the relationship between them.

The present study was part of a prospective multicenter study, in which 920 individuals (609 males, mean age, 62.78 ± 11.79 years) were included from November 2013 to December 2015. The cognitive function of the patients was evaluated, and the diagnosis of poststroke cognitive impairment (PSCI) was made during their stay in the hospital. A subgroup of 439 patients from a single center was followed up for 4 to 6 years and was reassessed for cognitive function.

According to the diagnostic criteria, the PSCI prevalence was lower in the fish-rich diet group (*P* < .05). After adjusting for demographic and clinical variables by logistic regression, patients with a habit of consuming a fish-rich diet had a lower risk of developing PSCI than patients without a fish-rich diet (odds ratio [OR]: 0.74; 95% confidence interval [CI]: 0.46–0.95). When MMSE score was considered the cognitive function outcome variable, the long-term cognitive function of the fish-rich diet group was better (28 [26–30] vs 27 [25–29], *P* < .01), but the statistical results were not significant after correcting for the related confounding factors (β: 0.13; 95% CI: −0.99–1.25; *P* = .82).

There was a negative relationship between consuming a fish-rich diet and the prevalence of PSCI, and there was no statistically significant difference in the relationship of a fish-rich diet on long-term cognitive function after stroke, which requires further study.

## 1 Introduction

Poststroke cognitive impairment (PSCI) is a common sequela of acute stroke and has been an important independent predictor of long-term adverse outcomes after stroke.^[1–4]^ The prevalence of PSCI ranges from 20% to 80% worldwide,^[5]^ and according to previous screening results, approximately 52.7% of patients with stroke develop PSCI in China.^[6]^ There is a north-to-south gradient in stroke prevalence in China; the annual incidence and mortality rate of stroke in the northeast region are highest, while those in the eastern coastal region are relatively lower.^[7]^ Vascular risk factors, including hypertension, insulin resistance, diabetes, obesity, hyperhomocysteinemia, and hyperlipidemia, are considered to be independent risk factors for the development of PSCI.^[8]^

As with the primary prevention of cerebrovascular diseases, healthy lifestyles such as a healthful diet and reasonable nutrition are related to better performance on cognition.^[9]^ High consumption of fish might be helpful to improve metabolic function, regulate blood pressure, and regulate blood lipids and could also prevent stroke, cardiovascular disease (CVD) and diabetes.^[10–12]^ Research has shown that the intake of fish may protect against all-cause dementia, including Alzheimer's disease (AD).^[13]^ Some observational studies have found that intake of fish may be related to a lower risk of AD and to slower cognitive decline.^[14,15]^ In short, eating fish is beneficial to the protection of cognitive functions, but there are still few studies directly concentrating on the relationship between fish and PSCI.

In the present study, we explored the relationship between a fish-rich diet and the prevalence of PSCI by logistic regression. Stratified analysis was conducted to examine whether the relationship was consistent across people with different characteristics. A 4- to 6-year follow-up was also carried out to evaluate the association between a fish-rich diet and long-term cognitive impairment. We present the following article in accordance with the STROBE reporting checklist.

## 2 Methods

### 2.1 Ethical approval

This study was approved by the Ethics Committee of the First Affiliated Hospital, College of Medicine, Zhejiang University (No. 2014-135) and was conducted in accordance with *the Declaration of Helsinki*. All subjects or their legal guardians signed informed consent forms.

### 2.2 Study design and participants

The present study was a cross-sectional study conducted to explore the relationship between a fish-rich diet and PSCI. This was part of a prospective multicenter cohort study designed to investigate the incidence and outcomes of patients with cognitive impairment after stroke. The trial was registered in the Chinese Clinical Trial Registry (registry number: ChiCTR-PRCH-14004805). This large-scale observational study, performed at seven Chinese medical centers, consisted of consecutive patients who received a confirmed diagnosis of acute stroke between November 2013 and December 2015. All of the centers are located in Zhejiang Province, East China.

**Figure 1:**
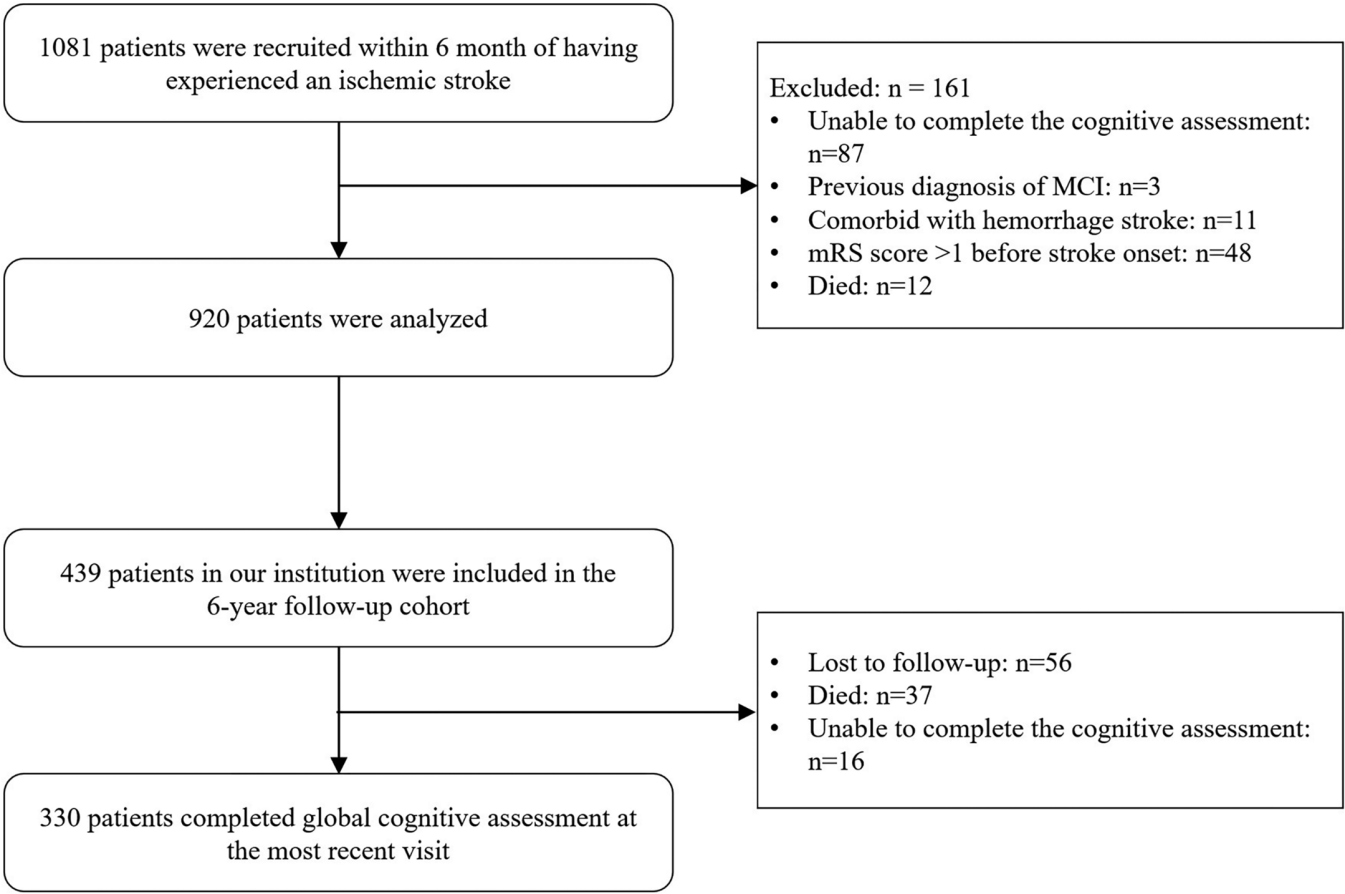
The study flow chart. A total of 920 patients were enrolled in the study; 439 patients were followed up, and follow-up data of 330 patients were included in the statistics. MCI = mild cognitive impairment, mRS = modified Rankin scale.

A subgroup of patients from a single center (the First Affiliated Hospital, College of Medicine, Zhejiang University) was followed up continuously for 4 to 6 years. Variable comparisons between the follow-up population and the overall population are shown in Table S1, Supplemental Digital Content. There was no significant difference between the two, indicating that the follow-up population could be considered representative. Cognitive assessment was performed again at follow-up. Information about patient deaths during follow-up, including the exact dates and causes of death, was determined by family members’ reports and medical records. The study flow chart is shown in Figure 1.

The following inclusion criteria were used to select patients eligible for inclusion in the present study: age ≥18 years and clinical diagnosis of acute ischemic stroke (AIS) according to the definition of stroke by the World Health Organization (WHO)^[16]^ and confirmed by computerized tomography (CT) or magnetic resonance imaging (MRI) within 6 months.

The exclusion criteria were as follows:

(1) inability to complete the assessment due to various reasons (including language barriers, aphasia, and consciousness disorder);
(2) a history of cognitive impairment before stroke;
(3) refusal to sign the informed consent;
(4) modified Rankin scale (mRS) score >1 before stroke;
(5) comorbid hemorrhagic stroke.

### 2.3 Demographic and clinical variables

Demographic characteristics, vascular risk factors, living habits, and other clinical characteristics were collected using questionnaires specifically designed for the cohort study at the time of enrollment.

Demographic characteristics included age, sex, and years of education. Vascular risk factors included hypertension (previous diagnosis of hypertension, blood pressure >140/90 mm Hg with random >3 times, or taking antihypertensive drugs), diabetes (previous diagnosis of diabetes, random blood glucose ≥ 11.1 mmol/L or fasting blood glucose (FBG) ≥ 7.0 mmol/L), history of hyperlipidemia, cerebrovascular disease and CVD, smoking (smoking ≥1 cigarette/day with duration of ≥1 year), drinking (drinking hard liquor at least once a week, drinking >50 mL, and duration of >6 months, or drinking mild liquor converted into alcoholic liquor according to its general alcohol content, in accordance with the above regulations), and body mass index (BMI).^[8]^ Related living habits included vegetable-rich diet (eating vegetables at least once a day), tea-drinking habit (drink tea at least four times per week), coffee-drinking habit (drink coffee at least four times per week) and regular exercise was (at least 30 minutes one to three times a week with sweating or significantly increased heart rate during exercise). The fish-rich diet was defined as eating fish more than five times a week.

The following clinical characteristics were also included in the present study: laboratory examinations (triglyceride and total cholesterol), intravenous thrombolysis, TOAST type (large-artery atherosclerosis [LAA], cardioembolism [CE], small-vessel occlusion lacunar [SVO], stroke of other determined etiology [SOE], stroke of other undetermined etiology [SUE]),^[16]^ Fazekas scales,^[17]^ National Institutes of Health Stroke Scale (NIHSS) score,^[18]^ and head MRI (the scan sequence included T1-weighted imaging, T2-weighted imaging, and diffusion-weighted imaging [DWI]). According to the head MRI results of the patients, the cerebral infarction site and the number of infarcts were recorded. The whole brain was divided into the following parts when the infarct site was recorded: the frontal lobe, parietal lobe, temporal lobe, occipital lobe, basal ganglia, thalamus, brainstem, cerebellum, and subcortical region (mainly including the radiative crown and the semidovale center).

### 2.4 Assessment and PSCI diagnosis

Assessments and PSCI diagnoses were carried out by evaluators who were all senior neurologists who had received standardized and unified training by professional neuropsychological evaluators in the First Affiliated Hospital, College of Medicine, Zhejiang University. Global cognitive status was evaluated at the time of enrollment.

In the present study, the term PSCI indicates all forms of cognitive disorder after a clearly identified incident ischemic stroke within 6 months, including vascular mild cognitive impairment (VaMCI) and vascular dementia (VaD). The diagnostic criteria include those listed by the American Heart Association/American Stroke Association (AHA/ASA)^[19]^ and the National Institute of Neurological Disorders and the Stroke-Association Internationale pour la Recherche et l’Enseignement en Neurosciences (NINDS-AIREN).^[20]^ Only patients diagnosed as cognitively normal according to both criteria were considered cognitively normal, while other patients were considered to have PSCI.

The clinical dementia rate (CDR) and the modified Rankin score (mRS) were assessed during the hospital stay. The CDR has been shown to be an excellent scale for assessing cognitive state and has been used at all stages of dementia.^[21,22]^ The mRS was also used to evaluate the degree of disability of patients.

The MMSE (mini-mental state examination) and mRS scores were assessed again at the 4- to 6-year follow-up among a subgroup from a single center, the First Affiliated Hospital of Zhejiang University, to evaluate the long-term outcomes of cognitive and physical function.

### 2.5 Statistical analysis

Counting data are expressed as numbers (%), while continuous data are expressed as medians (interquartile ranges [IQRs]) or means ± standard deviations (SDs). When comparing characteristics between two groups, the categorical variables were analyzed with the χ^2^ test; the continuous variables with a normal distribution were analyzed with the independent *t* test, and measurement data without a normal distribution were analyzed with the Mann–Whitney *U* test. Missing values were not processed during the statistical analysis.

Three unconditioned logistic regression models were gradually established; the dependent variable was whether the patient had been diagnosed with PSCI. Model I was the unadjusted model, showing the relationship between a fish-rich diet and PSCI without excluding the influence of any confounding factors. Model II was adjusted for demographic variables (including sex, age, and years of education). The adjustment factors of model III included the above factors and other clinical variables: BMI, smoking, drinking, tea-drinking habit, coffee-drinking habit, vegetable-rich diet, regular sports, hypertension, hyperlipidemia, diabetes, a history of AIS, blood total cholesterol, and blood triglycerides. The statistical results are expressed as odds ratios (ORs) and 95% confidence intervals (CIs).

A multivariate logistic regression model was used for the stratified analysis. The classification variables included age (≥65 years or not), sex (male or female), years of education (≤6 years or not), smoking (yes or no), drinking (yes or no), vegetable-rich diet (yes or no), regular sports (yes or no), BMI (≤23.9 or not), diabetes (yes or no), hypertension (yes or no), and hyperlipidemia (yes or no). The long-term effects of a fish-rich diet on cognitive recovery were calculated using a multiple linear regression, and the MMSE scores were used as outcome variables, with adjustment factors including sex, age, and years of education.

All statistical analyses were conducted using Statistical Product and Service Solutions v20.0 (SPSS, Inc., IBM Company, Chicago, IL). All statistical tests were two-tailed, and a value of *P* < .05 was considered significant.

## 3 Results

### 3.1 Patient characteristics

From November 2013 to December 2015, a total of 1081 consecutive participants with AIS were enrolled from seven medical centers in Zhejiang Province. According to the exclusion criteria, 161 patients were excluded for the following reasons: previous diagnosis of MCI (3 patients), comorbid hemorrhagic stroke (11 patients), failure to complete the cognitive assessment within 6 months after stroke (87 patients), death (12 patients), and an mRS score >1 before stroke onset (48 patients). Ultimately, 920 individuals (mean age, 62.78 ± 11.79 years) were recruited; 311 (34%) were female, and 609 (66%) were male (see flow chart, Fig. 1).

According to the diagnostic criteria, 526 patients were diagnosed with PSCI, and 394 patients had normal cognitive function. The prevalence of PSCI was 57.17% (95% CI: 53.97%–60.37%). The characteristics of the study population are described in Table 1. Compared with the normal control (NC) group, there were more females (*P* < .001) in the PSCI group. In addition, individuals in the PSCI groups were older (*P* < .05), less educated (*P* < .001), fatter (*P* < .01), drank more (*P* < .05), ate less fish (*P* < .01), and had more histories of AIS (*P* < .05), CVD (*P* < .001) and hyperlipidemia (*P* < .05).

**Table 1:** Characteristics of no fish-rich diet group and fish-rich diet group.

Variables	Total (n = 920)	No fish-rich diet (n = 661)	Fish-rich diet (n = 259)	*P*
Demographic characteristics				
Age (yr), mean ± SD	62.78 ± 11.79	62.66 ± 11.81	63.13 ± 11.74	.596
Male, n (%)	609 (66.20)	442 (66.87)	167 (64.48)	.513
Education (yr), median (IQR)	6 (4–9)	6 (3–9)	6 (6–9)	.010^∗^
Vascular risk factors				
BMI (kg/m^2^), mean ± SD	23.74 ± 3.21	23.71 ± 3.27	23.82 ± 3.02	.657
Smoking, n (%)	268 (29.13)	198 (29.38)	70 (27.03)	.785
Drinking, n (%)	320 (34.78)	245 (37.07)	75 (28.96)	.098
Hypertension, n (%)	597 (64.89)	435 (65.81)	162 (62.55)	.712
Diabetes, n (%)	237 (25.76)	174 (26.32)	63 (24.32)	.950
Hyperlipidemia, n (%)	91 (9.89)	64 (9.68)	27 (10.42)	.506
Arial fibrillation, n (%)	13 (1.41)	11 (1.66)	2 (0.77)	.352
TC (mmol/L), mean ± SD	4.31 ± 1.05	4.30 ± 1.05	4.35 ± 1.05	.505
TG (mmol/L), mean ± SD	1.37 (0.99–1.90)	1.34 (0.98–1.88)	1.46 (1.05–2.03)	.095
HDL (mmol/L), mean ± SD	1.05 (0.89–1.25)	1.06 (0.89–1.25)	1.02 (0.87–1.23)	.161
LDL (mmol/L), mean ± SD	2.45 (1.92–3.08)	2.48 (1.88–3.09)	2.43 (1.98–3.01)	.943
GLU (mmol/L), mean ± SD	5.81 ± 2.30	5.82 ± 2.24	5.80 ± 2.45	.911
Other related living habits				
Tea-drinking habit, n (%)	488 (53.04)	326 (49.32)	162 (62.55)	<.001^∗∗∗^
Coffee-drinking habits, n (%)	252 (27.39)	188 (28.44)	64 (24.71)	.285
Vegetable-rich diet, n (%)	701 (76.2)	490 (74.13)	211 (81.47)	.020^∗^
Regular sports, n (%)	348 (37.83)	241 (36.46)	107 (41.31)	.175
Stroke-related characteristics				
mRS score, median (IQR)	2 (1–3)	2 (1–3)	2 (1–3)	.204
NIHSS score, median (IQR)	2 (1–4)	2 (1–4)	2 (0–4)	.920
Intravenous thrombolysis, n (%)	47 (5.11)	41 (6.20)	6 (2.32)	.646
TOAST type, n (%)	n = 730			
LAA	61 (8.36)	47 (9.09)	14 (6.57)	
CE	489 (66.99)	343 (66.34)	146 (68.54)	.438
SVO	160 (21.92)	110 (21.28)	50 (23.47)	
SOE	10 (1.37)	8 (1.55)	2 (0.94)	
SUE	10 (1.37)	9 (1.74)	1 (0.47)	
Fazekas scale for white matter lesions (n = 906)				
Periventricular white matter, n (%)			<0.001^∗∗∗^	
0		223 (32.9)	154 (67.5)	
1		284 (41.9)	52 (22.8)	
2		121 (17.8)	16 (7.0)	
3		50 (7.4)	6 (2.7)	
Deep white matter, n (%)				<.001^∗∗∗^
0		330 (48.7)	164 (71.9)	
1		187 (27.6)	38 (16.7)	
2		103 (15.2)	19 (8.3)	
3		58 (8.6)	7 (3.1)	
Infarction location (%)				<.001^∗∗∗^
Frontal lobe		0.12	0.13	
Parietal lobe		0.11	0.11	
Temporal lobe		0.09	0.10	
Occipital lobe		0.07	0.08	
Basal ganglia		0.25	0.29	
Thalamus		0.07	0.08	
Brainstem		0.11	0.12	
CDR score, n (%)	n = 893			.022^∗^
0		228 (0.35)	112 (0.46)	.004^∗∗^
0.5		340 (0.52)	97 (0.39)	.001^∗∗^
1		66 (0.10)	29 (0.12)	.687
2		13 (0.02)	8 (0.03)	.383
PSCI, n (%)	526 (57.17)	401 (60.66)	125 (46.26)	.018^∗^

BMI = body mass index, CDR = clinical dementia rating, CE = cardioembolism, GLU = glucose, HDL = high density lipoprotein, IQR = interquartile range, LAA = large-artery atherosclerosis, LDL = low density lipoprotein, mRS = modified Rankin scale, NIHSS = National Institutes of Health Stroke Scale, PSCI = post-stroke cognitive impairment, SD = standard deviation, SOE = stroke of other determined etiology, SUE = stroke of undetermined etiology, SVO = small-vessel occlusion, TOAST = trial of org 10172 in acute stroke treatment, TC = total cholesterol, TG = triglyceride.

∗ *P* < .05.

∗∗ *P* < .01.

∗∗∗ *P* < .001.

**Figure 2:**
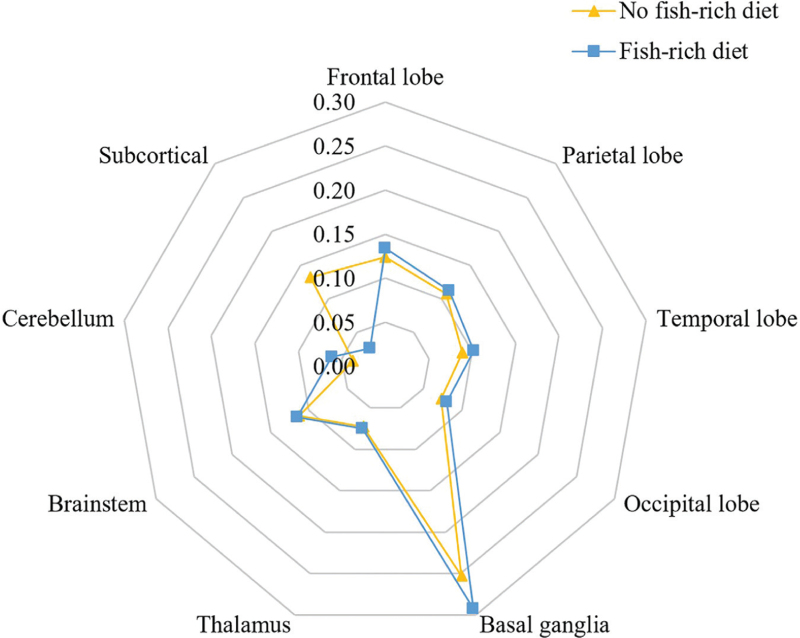
The distribution of infarcts of the no fish-rich diet group and the fish-rich diet group. According to the head MRI results of the patients, the cerebral infarction locations and the number of infarcts were recorded as: frontal lobe, parietal lobe, temporal lobe, occipital lobe, basal ganglia, thalamus, brainstem, cerebellum, and subcortical regions (mainly including the radiative crown and the semidovale center). The figure shows the number of infarcts in this brain region as a percentage of the total number of infarcts in all patients.

**Figure 3:**
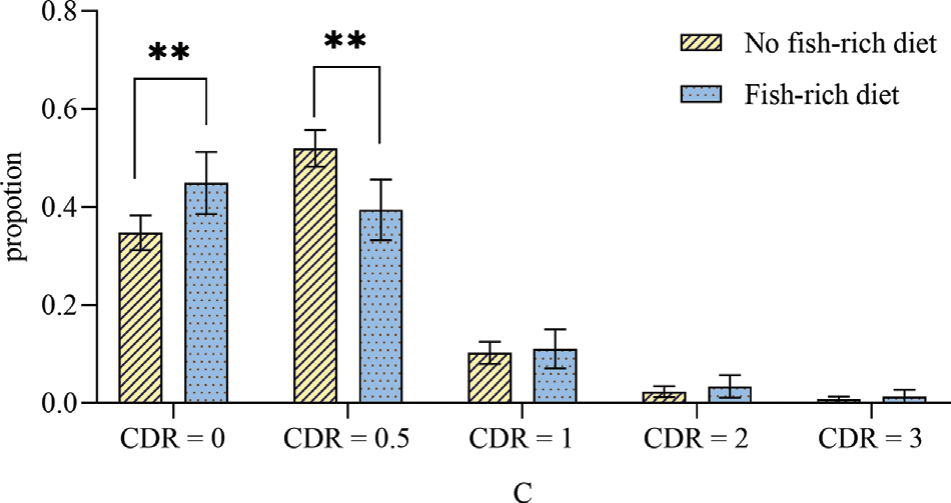
The distribution ratio of CDR scores of the no fish-rich diet group and the fish-rich diet group. CDR = clinical dementia rate; ∗∗*P* < .01.

Patients were divided into two groups according to whether they had a fish-rich diet (no fish-rich diet: 661 vs fish-rich diet: 259). The characteristics of the two groups were compared, and the results are shown in Table 1. In the fish-rich diet group, individuals were more educated (*P* < .05). They also had more tea-drinking habits (*P* < .001).and more vegetable-rich diet (*P* < .05). There were fewer white matter lesions (including paraventricular and deep white matter) (*P* < .001) and fewer subcortical infarctions (*P* < .001) in the fish-rich diet group (the distribution of infarcts of the two groups is shown in Fig. 2). The prevalence of PSCI in the fish-rich diet group was significantly lower than that in the no fish-rich diet group (*P* < .05). For the CDR score, the distribution of scores was significantly different between the two groups (*P* < .05). There was no significant difference in mRS scores between the two groups. The outcome of the scale assessment of the two groups is shown in Figure 3.

### 3.2 Analysis of the effect of the fish-rich diet

**Figure 4:**
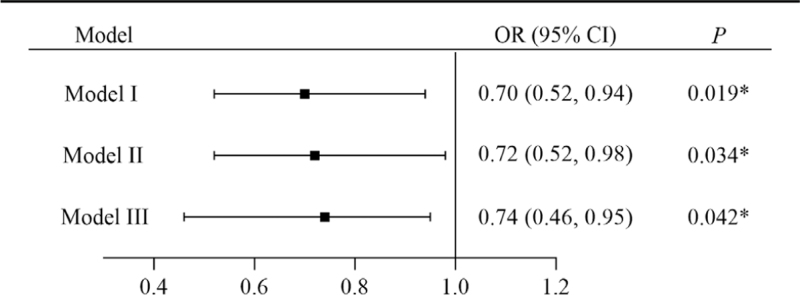
Effects of a fish-rich diet on PSCI, as analyzed by logistic regression models. Model I is the unadjusted model and shows the relationship with fish-rich diet and PSCI. Model II was adjusted for the demographic variables, including sex, age, and years of education. The adjustment factors of model III included the above factors and other clinical variables: BMI, smoking, drinking, tea-drinking habit, coffee-drinking habit, vegetable-rich diet, regular sports, hypertension, hyperlipidemia, diabetes, a history of AIS, blood total cholesterol, and blood triglycerides. AIS = acute ischemic stroke, BMI = body mass index, CI = confidence interval, OR = odds ratio, PSCI = poststroke cognitive impairment. ∗*P* < .05.

Three unconditioned logistic regression models were established gradually, while the outcome variable was set to determine whether PSCI occurred. Model I showed the relationship between a fish-rich diet and PSCI: OR: 0.70; 95% CI: 0.52–0.94. Model II was adjusted for sex, age, and years of education: OR: 0.72; 95% CI: 0.52–0.98). Model III was further adjusted for all possible confounding factors, including the above factors, BMI, smoking, drinking, tea-drinking habit, coffee-drinking habit, vegetable-rich diet, regular sports, hypertension, hyperlipidemia, diabetes, a history of AIS, blood total cholesterol, and blood triglycerides: OR: 0.74; 95% CI: 0.46–0.95 (as shown in Fig. 4). This showed that there was a significant relationship between the fish-rich diet and a lower prevalence of PSCI.

**Figure 5:**
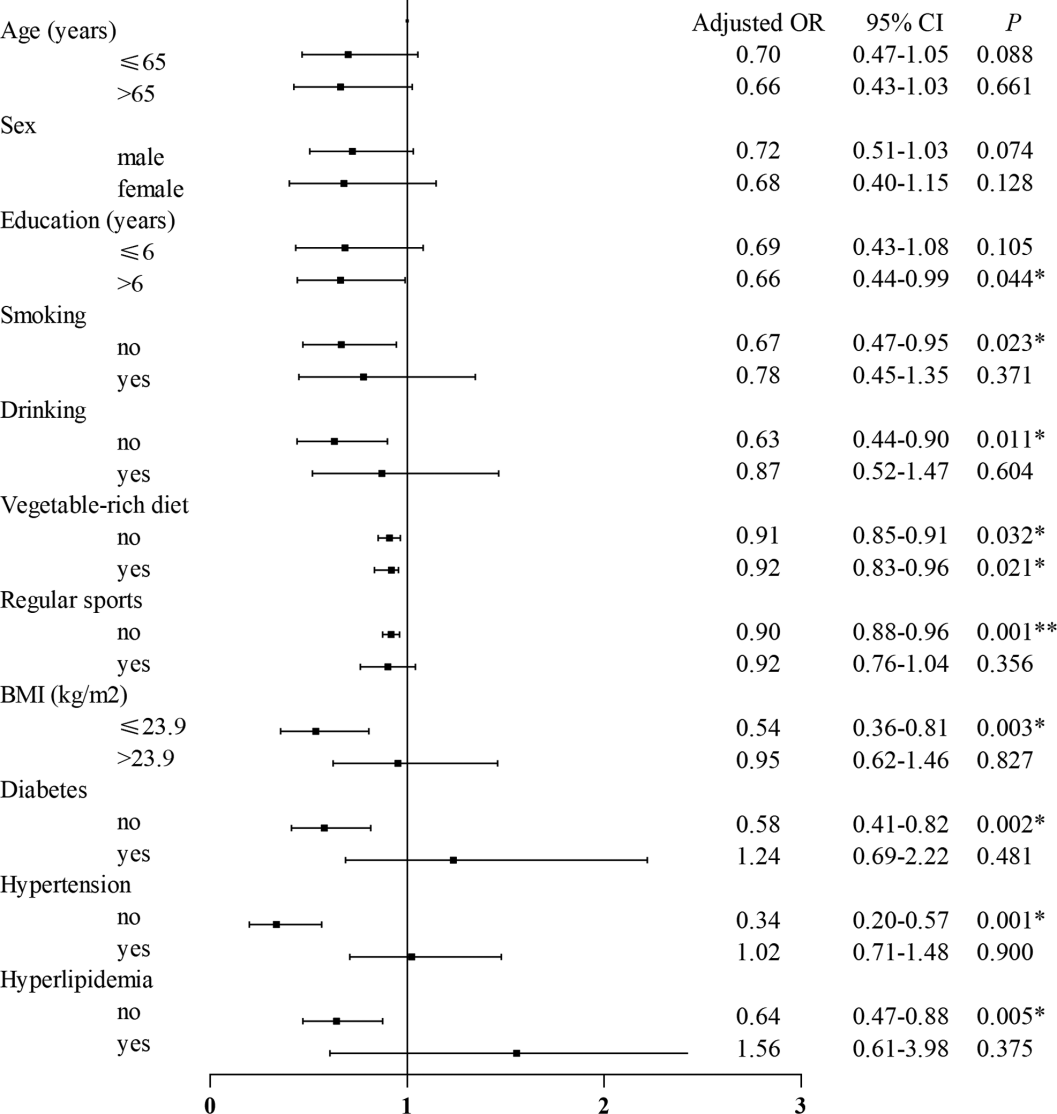
Forest plot of the stratified analysis of the effects of a fish-rich diet. BMI = body mass index, CI = confidence interval, OR = odds ratio. ∗*P* < .05, ∗∗*P* < .01.

Multivariate logistic regression analysis was used for stratified analysis of the effect of the fish-rich diet. We stratified the population according to some factors and analyzed the relationship between fish-rich diet and PSCI in different populations. The results showed that the fish-rich diet was associated with a lower prevalence of PSCI in the following conditions: years of education >6 (OR: 0.66; 95% CI: 0.44–0.99), no smoking (OR: 0.67; 95% CI: 0.47–0.95), no drinking (OR: 0.63; 95% CI: 0.44–0.90), BMI under 23.9 (OR: 0.54; 95% CI: 0.36–0.81), no diabetes (OR: 0.58; 95% CI: 0.41–0.82), no hypertension (OR: 0.34; 95% CI: 0.20–0.57), or no hyperlipidemia (OR: 0.64; 95% CI: 0.47–0.88) (as shown in Fig. 5).

### 3.3 Long-term effect of the fish-rich diet on cognitive function

The longitudinal follow-up results of the single center of the First Affiliated Hospital of Zhejiang University are shown in Table 2. A total of 439 patients in our institution were included in the 4- to 6-year follow-up cohort (average follow-up time, 57.53 ± 12.42 months), and 330 of them completed the cognitive assessment (as shown in Fig. 1). Among them, there were 205 males (62.12%), and 77 patients (23.33%) belonged to the fish-rich diet group. The MMSE scores of the fish-rich diet group and the no fish-rich diet group were compared between groups during follow-up, and the MMSE score of the fish-rich diet group was significantly higher than that of the no fish-rich diet group (28 [26–30] vs 27 [25–29], *P* < .01). Multiple linear regression was used to correct the influences of sex, age, and education, with the MMSE scores as outcome variables, of which the variable type was a continuous variable. After correcting for other factors, the fish-rich diet had no significant effect on cognitive function, suggesting that the difference in scores between the two groups may be a false positive caused by confounding factors (β: 0.13; 95% CI: −0.99 to 1.25; *P* = .82).

**Table 2:** Follow-up outcomes of no fish-rich diet and fish-rich diet.

	Total	No fish-rich diet	Fish-rich diet	*P*
Included in follow-up	n = 439	n = 334	n = 105	
Included in the analysis, n (%)	330 (75.17)	253 (75.75)	77 (73.33)	.617
Age (yr), mean ± SD	65.86 ± 12.03	66.22 ± 12.35	64.70 ± 10.77	.332
Male, n (%)	205 (62.12)	154 (60.87)	51 (66.23)	.369
Education (yr), median (IQR)	6 (4–9)	6 (3–9)	6 (6–9)	.027^∗^
mRS score, median (IQR)	1.00 (0.00–2.00)	1.00 (0.00–2.00)	1.00 (0.00–2.00)	.998
MMSE score, median (IQR)	27.00 (25.00–29.00)	27.00 (25.00–29.00)	28.00 (26.00–30.00)	.006^∗∗^

IQR = interquartile range, MMSE = mini-mental state examination, mRS = modified Rankin scale, SD = standard deviation.

∗ *P* < .05.

∗∗ *P* < .01, ^∗∗∗^*P* < .001.

## 4 Discussion

This prospective multicenter cross-sectional study included 1081 AIS survivors from seven medical institutions in Zhejiang Province, and 920 patients met the inclusion criteria. A 4- to 6-year longitudinal follow-up was carried out among the 439 patients at one center, of whom 330 completed a cognitive assessment at the end of the follow-up. The patient characteristics of the PSCI and NC groups were compared, and the relationship of the fish-rich diet and PSCI was explored by logistic regression. We found that a fish-rich diet was significantly related to a lower risk of PSCI after adjusting for the relevant demographic and clinical variables. There was no statistically significant difference in the effects of a fish-rich diet on long-term cognitive function after stroke, which requires further study in terms of expanding the sample size and further subgroup analysis.

In the present study, the prevalence of PSCI was 57.17%, which was similar to the averages of previous national and international studies.^[5,6]^ The differences might be due to different timing and diagnostic criteria for poststroke assessment. This study found that PSCI patients were older, had a low education level, and had more histories of TIA, CVD, and hyperlipidemia, which was similar to the results of previous studies,^[23–25]^ indicating that the common risk factors for stroke were significantly correlated with PSCI. In addition, compared with the NC group, the proportion of women in the PSCI group was higher, which was different from other studies.^[26,27]^ Whether this was related to the geographical locations and living habits of our patients remains to be further explored. The cognitive outcome of ischemic stroke was also associated with reperfusion therapies. There was a study showing that patients with endovascular treatment plus intravenous thrombolysis had better cognitive performance than those with intravenous thrombolysis alone.^[28]^ Since thrombectomy was not carried out in relevant hospitals during the study period, only some patients received thrombolysis in the present study. The relationship between fish-rich diet, reperfusion, and cognitive function after stroke may be further clarified by subsequent studies.

Most importantly, this study supported the protective effect of a fish-rich diet on the occurrence of PSCI. We confirmed the relationship between the fish-rich diet and lower PSCI by multivariable logistic regression. Several previous studies have shown the positive effect of a fish-rich diet on cognitive function. Fish consumption is cross-sectionally associated with a lower risk of dementia.^[14,15,29]^ In addition, the Mediterranean diet, as a diet mainly composed of unrefined cereals, vegetables, fruits, legumes, potatoes, fish and olive oil, has also received attention regarding its impact on cognitive function.^[30]^ Researchers have found that the Mediterranean diet has a protective effect on cognitive function^[31]^; fish are an important part of this diet.^[32]^ Fish intake is associated with a reduction in vascular risk factors, including lowering BMI and reducing the incidence of diabetes, hypertension, and hyperlipidemia.^[10,11,33]^ Eating fish is associated with a lower prevalence of subcortical infarcts and white matter abnormalities.^[34–36]^ This lower risk may be related to favorable effects of ingredients in fish diets, such as plasma phospholipid ω-3 polyunsaturated fatty acids (PUFAs) (e.g., docosahexaenoic acid (DHA), eicosapentaenoic acid (EPA) and other essential PUFAs), which have important effects on blood pressure, lipids, red blood cell deformability, inflammation, endothelial cell function, cerebral arteriolar reactivity, and platelet function.^[34]^ In our study, there was a significant difference in the distribution of stroke sites between the fish-rich diet group and the no fish-rich diet group, and the fish-rich diet group had fewer subcortical infarctions.

According to our results, the fish-rich diet was associated with a lower prevalence of PSCI in patients with more than 6 years of education and those with no smoking, no drinking, BMI under 23.9, no diabetes, no hypertension or no hyperlipidemia. A randomized controlled study showed that a 2-year multidomain intervention (diet, exercise, cognitive training, vascular risk monitoring) could improve or maintain cognitive functioning in at-risk elderly people from the general population.^[37]^ In summary, vascular risk factors and a fish-rich diet were both associated with PSCI. Diet is not a decisive factor in PSCI. To reduce the occurrence of PSCI, we need to comprehensively manage the overall risk factors for stroke and adopt a healthier lifestyle to have a better protective effect on cognitive function after stroke.

This study is subject to several limitations. First, in terms of diet plan, we only calculated statistics on whether patients could eat a fish-rich diet more than 5 days a week, whereas the method and amount of fish consumption were not recorded and analyzed in detail. The detail of other diet habits was not be recorded by professional dietary habit questionnaire. Second, we only suggested that the fish-rich diet before stroke had a protective effect on cognitive function, but in our long-term follow-up, no protective effect of a fish-rich diet on the brain was found. This may mean that there is a time limit to the protective effect of a fish-rich diet on cognitive function after stroke; further research is needed to confirm this possibility. As a prospective study, the selection centers of this study were all located in the southeast coastal area of China; thus, the representation of the whole dietary habits of China is limited. We speculate that populations in other parts of China will have lower proportions of fish-rich diets. A more detailed subgroup analysis is needed to screen the most effective components and mechanisms of cognitive effects in fish.

In conclusion, there was a negative relationship between consuming a fish-rich diet and the prevalence of PSCI, and there was no statistically significant difference in the effects of a fish-rich diet on long-term cognitive function after stroke, which requires further study.

## Acknowledgments

We are deeply appreciative of the participants in this study and their relatives and thank all staff of relevant centers (these centers included the First Affiliated Hospital of Zhejiang University, Zhejiang University Huzhou Hospital, Jinhua Municipal Central Hospital, Shaoxing People's Hospital, Ningbo Hospital of Zhejiang University, The First Hospital of Jiaxing, and The Second Hospital of Jiaxing) for their support and assistance.

## Author contributions

**Conceptualization:** Ben-Yan Luo, Jian Gao, Jiarui Li

**Data curation:** Fan-Xia Meng, Jiarui Li

**Formal analysis:** Fan-Xia Meng, Jiarui Li

**Funding acquisition:** Ben-Yan Luo

**Investigation:** Yang Yu

**Methodology:** Jiarui Li, Yang Yu

**Project administration:** Ben-Yan Luo

**Validation:** Ben-Yan Luo, Jian Gao

**Visualization:** Fan-Xia Meng

**Writing – original draft:** Jiarui Li

**Writing – review & editing:** Ben-Yan Luo, Fan-Xia Meng, Jian Gao, Yang Yu

## Supplementary Material

Supplemental Digital Content

## References

[1] Nys GM van Zandvoort MJ de Kort PL. The prognostic value of domain-specific cognitive abilities in acute first-ever stroke. Neurology 2005;64:821–7. 15753416 10.1212/01.WNL.0000152984.28420.5A

[2] Sibolt G Curtze S Melkas S. Poststroke dementia is associated with recurrent ischaemic stroke. J Neurol Neurosurg Psychiatry 2013;84:722–6. 23418214 10.1136/jnnp-2012-304084

[3] van der Zwaluw CS Valentijn SA Nieuwenhuis-Mark R Rasquin SM van Heugten CM. Cognitive functioning in the acute phase poststroke: a predictor of discharge destination? J Stroke Cerebrovasc Dis 2011;20:549–55. 20833083 10.1016/j.jstrokecerebrovasdis.2010.03.009

[4] Nys GM van Zandvoort MJ de Kort PL Jansen BP Kappelle LJ de Haan EH. Restrictions of the Mini-Mental State Examination in acute stroke. Arch Clin Neuropsychol 2005;20:623–9. 15939186 10.1016/j.acn.2005.04.001

[5] Sun J Tan L Yu J. Post-stroke cognitive impairment: epidemiology, mechanisms and management. Ann Transl Med 2014;2:80. 25333055 10.3978/j.issn.2305-5839.2014.08.05 PMC4200648

[6] Meng F Zhang S Yu J. Low hemoglobin levels at admission are independently associated with cognitive impairment after ischemic stroke: a multicenter, population-based study. Transl Stroke Res 2020;11:623–9. 10.1007/s12975-020-00785-1 32043214

[7] Wang W Jiang B Sun H. Prevalence, incidence, and mortality of stroke in China: results from a nationwide population-based survey of 480 687 adults. Circulation 2017;135:759–71. 28052979 10.1161/CIRCULATIONAHA.116.025250

[8] Sahathevan R Brodtmann A Donnan GA. Dementia, stroke, and vascular risk factors; a review. Int J Stroke 2012;7:61–73. 22188853 10.1111/j.1747-4949.2011.00731.x

[9] Reis JP Loria CM Launer LJ. Cardiovascular health through young adulthood and cognitive functioning in midlife. Ann Neurol 2013;73:170–9. 23443990 10.1002/ana.23836 PMC3608821

[10] He K. Fish, long-chain omega-3 polyunsaturated fatty acids and prevention of cardiovascular disease—eat fish or take fish oil supplement? Prog Cardiovasc Dis 2009;52:95–114. 19732603 10.1016/j.pcad.2009.06.003

[11] Panagiotakos DB Zeimbekis A Boutziouka V. Long-term fish intake is associated with better lipid profile, arterial blood pressure, and blood glucose levels in elderly people from Mediterranean islands (MEDIS epidemiological study). Med Sci Monit 2007;13:R307–12. 17599024

[12] Marushka L Batal M David W. Association between fish consumption, dietary omega-3 fatty acids and persistent organic pollutants intake, and type 2 diabetes in 18 First Nations in Ontario, Canada. Environ Res 2017;156:725–37. 28482294 10.1016/j.envres.2017.04.034

[13] Ozawa M Ninomiya T Ohara T. Dietary patterns and risk of dementia in an elderly Japanese population: the Hisayama study. Am J Clin Nutr 2013;97:1076–82. 23553168 10.3945/ajcn.112.045575

[14] Barberger-Gateau P Letenneur L Deschamps V Peres K Dartigues JF Renaud S. Fish, meat, and risk of dementia: cohort study. BMJ 2002;325:932–3. 12399342 10.1136/bmj.325.7370.932 PMC130057

[15] LopezLBKritz-SilversteinDBarrett-ConnorE. High dietary and plasma levels of the omega-3 fatty acid docosahexaenoic acid are associated with decreased dementia risk: the Rancho Bernardo study. J Nutr Health Aging 2011;15:25–31.2126751810.1007/s12603-011-0009-5

[16] Adams HJ Bendixen BH Kappelle LJ. Classification of subtype of acute ischemic stroke. Definitions for use in a multicenter clinical trial. TOAST. Trial of Org 10172 in Acute Stroke Treatment. Stroke 1993;24:35–41. 7678184 10.1161/01.str.24.1.35

[17] Fazekas F Chawluk JB Alavi A Hurtig HI Zimmerman RA. MR signal abnormalities at 1.5 T in Alzheimer's dementia and normal aging. Am J Roentgenol 1987;149:351–6. 3496763 10.2214/ajr.149.2.351

[18] Brott T Adams HP Olinger CP. Measurements of acute cerebral infarction: a clinical examination scale. Stroke 1989;20:864–70. 2749846 10.1161/01.str.20.7.864

[19] Gorelick PB Scuteri A Black SE. Vascular contributions to cognitive impairment and dementia: a statement for healthcare professionals from the American Heart Association/American Stroke Association. Stroke 2011;42:2672–713. 21778438 10.1161/STR.0b013e3182299496 PMC3778669

[20] Roman GC Tatemichi TK Erkinjuntti T. Vascular dementia—diagnostic-criteria for research studies—report of the NINDS-AIREN International Workshop. Neurology 1993;43:250–60. 8094895 10.1212/wnl.43.2.250

[21] Diehl J Kurz A. Frontotemporal dementia: patient characteristics, cognition, and behaviour. Int J Geriatr Psychiatry 2002;17:914–8. 12325050 10.1002/gps.709

[22] Vas CJ Pinto C Panikker D. Prevalence of dementia in an urban Indian population. Int Psychogeriatr 2001;13:439–50. 12003250 10.1017/s1041610201007852

[23] Farooq MU Gorelick PB. Vascular cognitive impairment. Curr Atheroscler Rep 2013;15:330. 23612956 10.1007/s11883-013-0330-z

[24] Zou Y Zhu Q Deng Y. Vascular risk factors and mild cognitive impairment in the elderly population in Southwest China. Am J Alzheimers Dis Other Demen 2014;29:242–7. 24375574 10.1177/1533317513517042 PMC10852946

[25] Lu D Ren S Zhang J Sun D. Vascular risk factors aggravate cognitive impairment in first-ever young ischaemic stroke patients. Eur J Neurol 2016;23:940–7. 26917058 10.1111/ene.12967

[26] Li J Wang J Wu B. Association between early cognitive impairment and midterm functional outcomes among Chinese acute ischemic stroke patients: a longitudinal study. Front Neurol 2020;11:20. 32174878 10.3389/fneur.2020.00020 PMC7054458

[27] Suda S Nishimura T Ishiwata A. Early cognitive impairment after minor stroke: associated factors and functional outcome. J Stroke Cerebrovasc Dis 2020;104749. 32178931 10.1016/j.jstrokecerebrovasdis.2020.104749

[28] Lattanzi S Coccia M Pulcini A. Endovascular treatment and cognitive outcome after anterior circulation ischemic stroke. Sci Rep 2020;10:18524. 33116220 10.1038/s41598-020-75609-1 PMC7595128

[29] Anastasiou CA Yannakoulia M Kosmidis MH. Mediterranean diet and cognitive health: initial results from the Hellenic Longitudinal Investigation of Ageing and Diet. PLoS ONE 2017;12:e182048. 10.1371/journal.pone.0182048 PMC5538737 28763509

[30] Aridi YS Walker JL Wright ORL. The association between the Mediterranean dietary pattern and cognitive health: a systematic review. Nutrients 2017;9:674. 10.3390/nu9070674 PMC5537789 28657600

[31] van den Brink AC Brouwer-Brolsma EM Berendsen AAM van de Rest O. The Mediterranean, Dietary Approaches to Stop Hypertension (DASH), and Mediterranean-DASH Intervention for Neurodegenerative Delay (MIND) diets are associated with less cognitive decline and a lower risk of Alzheimer's disease—a review. Adv Nutr (Bethesda, Md) 2019;10:1040–65. 10.1093/advances/nmz054 PMC6855954 31209456

[32] Qin B Adair LS Plassman BL. Dietary patterns and cognitive decline among Chinese older adults. Epidemiology (Cambridge, Mass) 2015;26:758–68. 10.1097/EDE.0000000000000338 PMC5928777 26133024

[33] Fotuhi M Mohassel P Yaffe K. Fish consumption, long-chain omega-3 fatty acids and risk of cognitive decline or Alzheimer disease: a complex association. Nat Clin Pract Neurol 2009;5:140–52. 19262590 10.1038/ncpneuro1044

[34] Virtanen JK Siscovick DS Longstreth WJ Kuller LH Mozaffarian D. Fish consumption and risk of subclinical brain abnormalities on MRI in older adults. Neurology 2008;71:439–46. 18678827 10.1212/01.wnl.0000324414.12665.b0 PMC2676980

[35] Huang TL Zandi PP Tucker KL. Benefits of fatty fish on dementia risk are stronger for those without APOE epsilon4. Neurology 2005;65:1409–14. 16275829 10.1212/01.wnl.0000183148.34197.2e

[36] Mozaffarian D Longstreth WJ Lemaitre RN. Fish consumption and stroke risk in elderly individuals: the cardiovascular health study. Arch Intern Med 2005;165:200–6. 15668367 10.1001/archinte.165.2.200 PMC1201399

[37] Ngandu T Lehtisalo J Solomon A. A 2 year multidomain intervention of diet, exercise, cognitive training, and vascular risk monitoring versus control to prevent cognitive decline in at-risk elderly people (FINGER): a randomised controlled trial. Lancet 2015;385:2255–63. 25771249 10.1016/S0140-6736(15)60461-5

