# The relationship between a fish-rich diet and poststroke cognitive impairment: A cross-sectional study with a follow-up in China: Erratum

**DOI:** 10.1097/MD.0000000000030548

**Published:** 2022-09-02

In the article, “The relationship between a fish-rich diet and poststroke cognitive impairment: A cross-sectional study with a follow-up in China,”^[1]^ which published in Volume 101, Issue 25 of *Medicine*, Dr Jie Yu has been removed from the author list at the authors’ request.
