# Supplementary material for: The relationship between a fish-rich diet and poststroke cognitive impairment: A cross-sectional study with a follow-up in China
Source: Medicine (Baltimore). 2022 Jun 24;101(25):e29234. doi: 10.1097/MD.0000000000029234 (PMC9276365; doi:10.1097/MD.0000000000029234)
Supplement: Supplemental Digital Content [file medi-101-e29234-s001.docx]

| **Supplemental Table 1: Comparison of demographic variables between the total population and the follow-up subgroup** | | | |
| --- | --- | --- | --- |
|  | Total | Follow-up group | *P* |
|  | n = 920 | n = 330 |  |
| Age (years)^1^, mean ± SD | 62.78 ±11.79 | 61.82 ± 11.43 | 0.786 |
| Male, n (%) | 609 (66.20) | 205 (62.12) | 0.183 |
| Education (years), median (IQR) | 6 (4- 9) | 6 (4- 9) | 0.856 |
| Fish-rich diet, n (%) | 259 (28.15) | 77 (23.33) | 0.347 |
| ^1^ Age refers to the age of baseline. | | | |
